# Endoscopic ultrasound gastroenterostomy: how to avoid inadvertent gastrocolostomy

**DOI:** 10.1055/a-2234-3953

**Published:** 2024-02-02

**Authors:** Jimil Shah, Abhirup Chatterjee, Vaneet Jearth, Anupam K. Singh

**Affiliations:** 1 Department of Gastroenterology, Postgraduate Institute of Medical Education and Research, Chandigarh, India

Endoscopic ultrasound (EUS)-guided gastroenterostomy (EUS-GE) is a commonly used modality in patients with gastric outlet obstruction, with results equivalent to surgical gastrojejunostomy 1 2 . Inadvertent gastrocolostomy is a rare but major complication of EUS-GE 3 . As the transverse colon is a mobile structure, it can overlap the jejunal loop, and failure to identify this can lead to inadvertent gastrocolostomy 3 .

A 76-year-old man with metastatic pancreatic carcinoma presented with symptoms of gastric outlet obstruction. After multidisciplinary team discussion, the patient was scheduled for EUS-GE using a device-assisted method (nasojejunal catheter). On EUS examination, after liberal instillation of methylene blue stained normal saline within the small bowel, distended jejunal loops could be visualized. However, on careful examination, a different bowel loop could be seen between the gastric wall and the jejunal loop. The overlapping bowel loop had three features that differed from the jejunal loops: absence of valvulae conniventes, presence of solid/semisolid floating material within the lumen (brown arrow in Fig. 1 **b,c** ), and absence of the intraluminal nasojejunal catheter. In contrast, jejunal loops had valvulae conniventes (blue arrow in Fig. 1 **a,c** ), clean fluid within the lumen (white arrow in Fig. 1 **a** ), and the intraluminal nasojejunal tube could be seen. Based on these features, we diagnosed a colonic overlapping loop between the gastric wall and jejunal loops. In view of this overlapping colonic loop, safe access to the jejunal loop could not be secured ( Fig. 1 , Video 1 ). The procedure was abandoned and the patient continued nasojejunal tube feeding for the next 48 hours. After 48 hours, successful EUS-GE was performed.

**Fig. 1:**
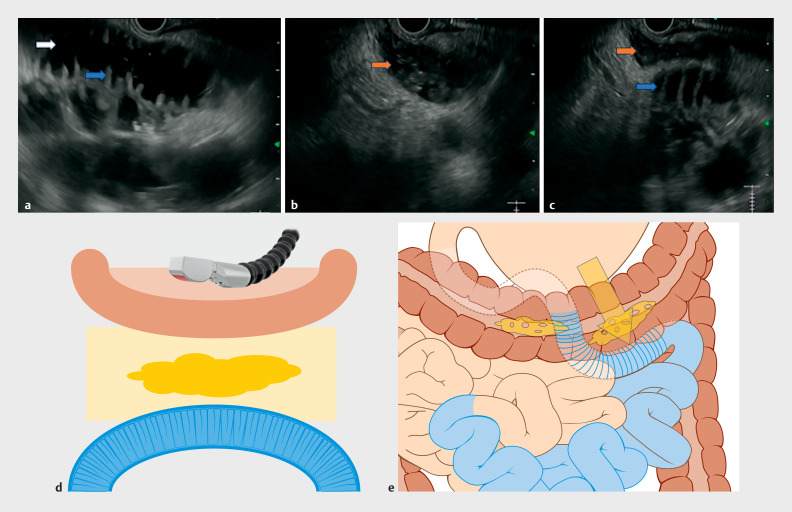
Features of the intestinal structures on endoscopic ultrasound. **a** Dilated jejunal loops after instillation with methylene blue-stained normal saline. The jejunal loops could be identified by the presence of valvulae conniventes (blue arrow) and clean fluid within the lumen (white arrow). **b** The colonic loop was identified by absence of valvulae conniventes and presence of solid fecal material within the colon (brown arrow). **c,d,e** Colonic loop coming between the gastric wall and the jejunal loop throughout the length of the dilated jejunal loops. With this configuration, safe access for gastrojejunostomy could not be secured. **e** Close up view of yellow framed area in **d** . Yellow arrow, planned route of gastroenterostomy.

Endoscopic ultrasound (EUS) examination of an overlapping colonic loop between the gastric wall and jejunal loop during EUS-guided gastroenterostomy.Video 1

In this case, we believe that due to liberal instillation of normal saline, the transverse colonic loop became distended with fluid and mobilised between the gastric wall and jejunum. We did not puncture the colonic loop as might have been expected to be filled with methylene blue-stained normal saline 3 . This case highlights important characteristics of colonic loops on EUS examination and the steps needed to identify these features to prevent inadvertent gastrocolostomy.

Endoscopy_UCTN_Code_TTT_1AS_2AB

## Notes

Endoscopy E-Videos https://eref.thieme.de/e-videos
*E-Videos* is an open access online section of the journal *Endoscopy* , reporting on interesting cases and new techniques in gastroenterological endoscopy. All papers include a high-quality video and are published with a Creative Commons CC-BY license. Endoscopy E-Videos qualify for HINARI discounts and waivers and eligibility is automatically checked during the submission process. We grant 100% waivers to articles whose corresponding authors are based in Group A countries and 50% waivers to those who are based in Group B countries as classified by Research4Life (see: https://www.research4life.org/access/eligibility/ ). This section has its own submission website at https://mc.manuscriptcentral.com/e-videos .
